# Survey on the management of childhood epilepsy among general practitioners in the area of Marrakech

**DOI:** 10.1186/s12887-023-03947-w

**Published:** 2023-04-04

**Authors:** Widad Lahmini, Samuel Opoku Gyamfi, Mounir Bourrous

**Affiliations:** Department of Paediatric Emergency, Mohamed VI UHC, Cadi Ayyad University, Faculty of Medicine and Pharmacy, PO Box: 7010, Sidi Abbad Street, 40000 Marrakech, Morocco

**Keywords:** Epilepsy, Child, Management, General practitioners, Survey

## Abstract

**Background:**

Epilepsy is a common chronic neurological disorder in the pediatric population and its evolution can be fatal. It represents a major public health problem as well as an economic burden for the families of affected children, health systems and the overall economies of countries. This further accentuates the role that general practitioners can play in the management of childhood epilepsy in the face of the persistent lack of neurologists and neuro-pediatricians in our country.

**Methods:**

In order to assess the knowledge and therapeutic habits of general practitioners, we carried out a descriptive and cross-sectional study with general practitioners practicing in the two healthcare sectors: public and private, and in two settings: urban and rural, during the year 2018. The data was collected through a pre-established survey.

**Results:**

In total, 155 general practitioners responded to the survey. For 85.2% of physicians, the diagnosis of childhood epilepsy was based on interrogation, physical examination, and EEG. While brain imaging would be systematic regardless of the type of epilepsy for 45.2% of doctors. Only 6 doctors (3.9%) had knowledge of the latest classifications of the “ILAE”. For treatment, the majority of physicians (65.5%) adopted first-line monotherapy with valproate in leading position. Almost half of the doctors (48.4%) found that education of parents and children was always necessary. None of the GPs interviewed in our series assessed the academic impact of epilepsy. Only 32% of doctors had received continuing education on epilepsy.

**Conclusion:**

The data from our study demonstrates that continuing education on the management of childhood epilepsy and the greater involvement of general practitioners were essential elements in improving care.

**Supplementary Information:**

The online version contains supplementary material available at 10.1186/s12887-023-03947-w.

## Introduction

Childhood epilepsy is a real public health problem. It represents an economic burden on the families of affected children, the health systems and the overall economy especially in low-income countries [1]. This disease continues to suffer from insufficient care, especially in developing countries. This is partly due to the lack of the material and financial resources necessary to manage the disease, and the lack of sufficiently trained medical and paramedical staff to properly manage childhood epilepsy [2]. This further emphasizes the role that general practitioners (GPs) can play in our health system, and the importance of providing them with all the necessary skills and involvement for better management of childhood epilepsy in our context.

To approach GPs' medical practices in childhood epilepsy, we conducted a survey in the Marrakech region, which turns out to be the first of its kind to be carried out in Morocco. Its objectives were to assess the knowledge of general practitioners in matters of childhood epilepsy, their treatment habits and to assess the gap with the recommendations.

## Methods

This was a descriptive and cross-sectional survey of general practitioners to assess their daily practices in the care of children followed for epilepsy. This study took place for 06 months during the year 2018. The target population was made up of GPs from the Marrakech region working in the two sectors: public (hospital, health center) and private (doctor's office, clinic). In total, a sample of 200 GPs were interviewed in our survey. Only 155 (77.5%) physicians agreed to complete the pre-established questionnaire.

Our study assessed the knowledge and therapeutic habits of GPs in the Marrakech region about childhood epilepsy. Doctors were questioned on the following points: the incidence and prevalence of childhood epilepsy, the most affected age group, the clinical and para-clinical diagnosis, the differential diagnoses, the factors triggering the epileptic seizures, the main causes, the recommendations of the International League Against Epilepsy "ILAE" [3], the treatment of the seizure and the basic treatment, education and psychological support, as well as follow-up and surveillance of the children. We also asked for their suggestions to improve the care of children followed for epilepsy.

The data were collected through a questionnaire distributed according to the following formula in order to reduce researcher bias, and to obtain a harmonised understanding of all the interviewed doctors: a single doctor distributed the questionnaires to general practitioners in both sectors, private and public on the basis of anonymity and confidentiality. This questionnaire consisted of 31 questions, the majority of which were of closed type, assessing the knowledge and therapeutic habits of practitioners in matters of childhood epilepsy. Filling of the questionnaire was previously conditioned by the consent of the doctors. The survey data was entered into Microsoft Excel software. Data analysis was carried out using Epi-info version 6 software at the Epidemiology Department of the Faculty of Medicine and Pharmacy of Marrakech.

## Results

Among the 155 physicians, 74.8% worked in the public sector and 64.5% in urban areas. With regards to the duration of medical practice, 45.5% of physicians had more than 10 years of experience. Only 49 doctors (31.6%) said they received further training on the management of epilepsy in children. Regarding the frequency of epilepsy in children, most doctors (65.8%) admitted that it is frequent, while 32 doctors (20.6%) replied that it is a rare pathology. The majority of physicians surveyed (80%) reported fewer than 5 cases of childhood epilepsy per month in their workplaces, while 9.7% reported between 5 to 10 cases per month. The age groups most encountered by physicians were those between 2 to 6 years and 6 to 12 years reported by 44.5 and 29.6% of physicians respectively.

**Fig. 1:**
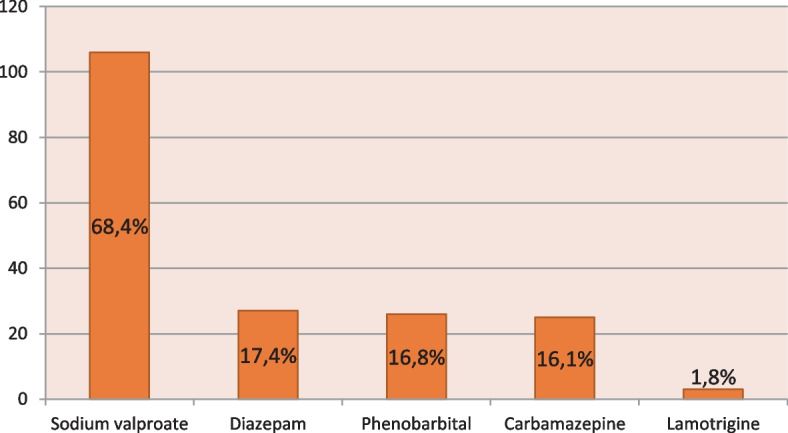
Distribution of antiepileptic molecules prescribed by the GPs

The majority of doctors (60%) found childhood epilepsy to be a serious condition. Just 6 doctors (3.9%) in our survey had knowledge of the latest classifications of the “ILAE” (Table 1). On the other hand, only 24 doctors (15.5%) said that they dealt with cases of childhood epilepsy individually and without the help of specialists. The rest of the doctors (84.5%) systematically referred them to specialists. The genetic etiologies were the most described, reported by the majority of physicians (66.5%). The other causes reported were: infectious (31%), traumatic (15.5%), lesional (29%) and tumoral (8.4%). The idiopathic etiology was described by 37.4% of physicians. According to the doctors surveyed, the main factors triggering seizures were: fever (74.2%), stress (62%), lack of sleep (50.3%), and stimulating drinks (25.2%). Heatstroke was reported by 23.2% of physicians. For the diagnosis of the disease, the majority (85.2%) declared reliance on the electro-clinical confrontation, by the realization of a systematic electroencephalogram (EEG). For 45.2% of physicians, brain imaging (CT-Scan or MRI) is routine regardless of the type of epilepsy. For the diagnosis of specific cases in our survey, 93 physicians (60%) had never diagnosed focal epilepsy in a child. Compared to the duration of the seizure necessary to make the diagnosis of status epilepticus in children faced with a persistent seizure or repeated seizures at close range, more than a third of the doctors questioned (36.1%) reported a duration of 15 minutes (Table 2). More than two-thirds of physicians surveyed (71.6%) said they rule out differential diagnoses before making a diagnosis of childhood epilepsy. The main differential diagnoses mentioned by the doctors questioned were: hypoglycemia (18.7%), febrile convulsions (17.4%), purulent meningitis (11.6%), brain tumor (7.7%). Concerning the therapeutic management, 102 doctors (65.8%) adopted the monotherapy, against 32 (20.6%) who were for the dual therapy in first intention. Regarding the duration of the basic treatment most of GPs opted for 2 years (33.5%). For the choice of molecules, the vast majority of doctors questioned (68.4%) opted for Sodium Valproate (Fig. 1). Most doctors (75%) were looking for side effects. According to the doctors interviewed, the most difficult syndromes to manage were: infantile spasms mentioned by 52 doctors (33.5%), tonic-clonic epilepsy reported by 52 doctors (33.5%), absence epilepsy noted by 37 physicians (23.9%) and myoclonic epilepsy (14.8%). In our survey, the majority of physicians (76.8%) said they followed up children after the first consultation. For the psychological care of children followed for epilepsy, more than two-thirds of doctors (39.4%) found that its indication was systematic. Nearly half of the physicians (48.4%) felt that the education of parents and children was still necessary. None of the GPs interviewed in our series assessed the academic impact of epilepsy.

The main recommendations of the interviewed doctors (51.6%) for improving the management of epilepsy in children were : the organization of continued training sessions for GPs on epilepsy in children, educating parents and/or those around them on childhood epilepsy, and improving coordination between GPs and specialists (neurologists, pediatric neurologists).

## Discussion

Epilepsy is the most common chronic neurological pathology in children. It affects 0.5 to 1% of children worldwide, and one in 150 children has epilepsy during the first 10 years of life [4]. The incidence is highest in low to middle income countries [5]. Indeed, nearly 80% of people with epilepsy live in these countries [2]. In children, the incidence of epilepsy is highest in the first year of life and decreases in adulthood from the age of 10 years [5]. World Health Organization (WHO) also draws attention to the fact that treatment for epilepsy is very rarely available in low-income countries. For Morocco, we have little information on this subject because very few studies have been published on this subject [6]. A study carried out in Marrakech in 2010 estimated that cases of childhood epilepsy represented 8.5% of pediatric service consultants [7]. In Australia, a similar survey found that GPs see a median of six patients with epilepsy, mostly adults [8]. In another Australian study, childhood epilepsy (0–14 years) accounted for 6.6% of all epilepsy treated with MG [9]. While the International Bureau of Epilepsy carrying out a survey in 16 different countries (Europe, Canada, USA and Asia) showed that 27% of the doctors questioned encountered at least 4 cases of childhood epilepsy per day, and almost half of the doctors saw an average of 1 case per day [10]. These consultation rates remain high compared to our survey where the majority of the interviewed doctors (80%) encountered less than 5 cases of childhood epilepsy per month in their workplaces. According to statistics from the WHO, Africa has the lowest rates of health personnel per capita resulting in reduced access to basic and quality health care [2]. In Morocco, GPs are at the forefront of the care of patients followed for epilepsy given the lack of pediatricians and neuro-pediatricians. According to a Swedish study, more than a third of children (36.6%) did not have access to the neuropediatrician [11]. In our survey, two-thirds of GPs surveyed (67.8%) had never benefited from continued education sessions about childhood epilepsy. This explains the lack of knowledge of the latest classification of the ILAE (3.9%) and the large number of transfers to specialists (84.5%) in our survey. These data once again emphasize the value of involving and training GPs for better management of childhood epilepsy. A study conducted in Brazil showed that the majority of general practitioners and pediatricians (72.2%) were unsatisfied with their further education on childhood epilepsy [12]. While in Australia, only 42% considered their knowledge of epilepsy adequate for their practice [13]. Another study carried out in Laos found that only a minority of GPs (3.5%) remembered having received training on epilepsy and concluded that improving their knowledge will go a long way towards better management of epilepsy [14]. In Latin America, an initiative to address the shortage of neurologists through e-learning for the benefit of GPs was concluded with satisfactory results in terms of adherence to courses with considerable improvement in their knowledge for better management of childhood epilepsy [15]. Likewise, a survey carried out in Zambia had shown that through a pilot project, a targeted training program can improve the knowledge and confidence of health workers towards better management of childhood epilepsy [16].

The etiologies of epilepsies are multiple and varied resulting from the conjunction of genetic and acquired factors. Idiopathic epilepsies are strongly correlated with a genetic predisposition. The etiology is significantly correlated with the age of onset. About half have a documented etiology of which about 22% are presumed to be of genetic origin [5]. Two thirds of GPs (66.5%) cited genetic etiologies as the main causes of childhood epilepsies. In developed countries, the vast majority of cases are currently not preventable. In contrast, in developing countries, perinatal causes, central nervous system infections and head trauma are predominant [5, 17, 18]. According to an African study, perinatal accidents are the main causes in children (10.8%) [19]. Harimanana’s study found that 56.3% of GPs did not know any cause for the epilepsy [14]. Locally, the study carried out in the Marrakech region in 2010 found that the etiologies were dominated by perinatal complications (31.6%) [7]. During our investigation, the lesional etiologies were mentioned by 29% of physicians surveyed. Misdiagnosis of epilepsy is common, especially in pediatrics. Thus, the attending physician should take the necessary precautions to avoid overdiagnosis sparing the child and his family unnecessary treatment and lifestyle restrictions. A Danish study found that over a third of referred children (39%) did not suffer from epilepsy [20]. Another Norwegian study showed that 32% of diagnostic errors were due to a false judgment of treating physicians [4]. In Laos, only 50.7% of GPs mentioned differential diagnoses [14]. In our survey, this rate was 71.6%. However, it turned out that the vast majority (96%) of GPs referred to differential diagnoses other than those reported in the literature. While EEG video is currently the gold standard that should be used in questionable cases, the use of smartphones in these situations should be encouraged in low-income countries, allowing better description and classification of types of crises [21, 22]. A large majority of our GPs (85.2%) said they rely on performing a systematic EEG to support the diagnosis and none mentioned the usefulness of smartphones. Regarding therapeutic management, two-thirds of our GPs (65.8%) adopted monotherapy, which complies with the ILAE recommendations. Sodium Valproate also remains the antiepileptic of choice in our survey (68.4%). This can be explained by lack of continued education sessions about childhood epilepsy, absence of actualisation of GPs’ knowledge about the new antiepileptics leaving their prescriptions classical. So, for most GPs, VPA is the drug of choice for many pediatric epileptic syndromes. Nevertheless, phenobarbital was described by 16.8% of GPs in our series against 33% by Harimanana and al. [14]. Generally, newly diagnosed patients with epilepsy are initially treated with monotherapy. Polytherapy should be considered only after the failure of at least two or three monotherapies in case of pharmacoresistant epilepsy [23]. Most of our GPs (65.8%) opted for the monotherapy. Concerning the side effects of treatment, most GPs in our series were warned about this problem (75%). This was not the case in other surveys like Gomes's study [12] in which most practitioners (93.3%) are not sensitized on this problem. A very recent study among pediatric patients aged 6–18 years showed that 37% of them described different adverse drug events dominated by neurologic or psychiatric symptoms [24]. Most of the studies emphasize the importance of normal schooling for better integration of children treated for epilepsy. Active communication between medical institutions and the school with clearly provided information is necessary for better safety of pupils being monitored for epilepsy at school [25]. School performance difficulties (SPD) in children with seizures are probably related to the neurobiological, cognitive, psychological, and social consequences of seizure recurrence [5]. Thus, children with recurrent seizures or epilepsy had a higher rate of SPD than children without seizures [26]. None of the physicians interviewed in our series assessed the educational impact of epilepsy. A very recent study showed that children with epilepsy may exhibit visuospatial memory impairment compared to their peer. These disorders may be correlated to some features of the epilepsy itself and to the impairment of executive functions. Different antiseizure medications can affect visuospatial memory differently, so it is important monitoring this aspect in pediatric patients [27]. The development of cognitive and behavioral side effects had been also reported with the new antiepileptic drugs in pediatric epilepsy [28]. Moreover, Perampanel in association with 1 or 2 concomitant antiseizure medications demonstrated a good effectiveness in pediatric patients, without having to use a high dose of the drug [29]. The psychological and social care of the child with epilepsy and his parents is also an essential part of the treatment. Indeed, epilepsy is a major source of anxiety and depression for children and their parents [21, 30]. These comorbidities potentially affect seizure control with repercussions on the prognosis and quality of life of children [21]. In a cross-sectional observational study, Operto et al. [31] found in the epilepsy pediatric group a higher levels of parental stress and higher presence of emotional and behavioral symptoms compared to controls, mainly represented by internalizing problems (anxiety and depression symptoms). Therefore, parents of children with epilepsy should be offered psychological support to cope with stress and to improve the relationship with their children [31]. Only 39.4% of GPs in our survey considered this psychotherapy to be always necessary. Therapeutic education of the family and the child followed for epilepsy is an essential part of management. This participatory role of parents will contribute to good adherence to treatment, better monitoring of the social life of their children thus avoiding excessive restrictions negatively impacting the psychosocial development of children. It was only performed in only half of our GPs (48.4%) compared to 72.2% in Harimanana’s study [14].

## Conclusion

Our study showed a lack of knowledge on the part of general practitioners regarding the management of epilepsy in children. It has also pinpointed erroneous attitudes towards international recommendations; but also their enthusiasm to improve their skills in this field. This highlights the value of a targeted training program adapted to our context. This can only be very beneficial for better management and improvement in the management of childhood epilepsy

## Supplementary Information

**Additional file 1: Supplementary Material 1.**

## Data Availability

The datasets used and/or analyzed during the current study are available from the corresponding author upon reasonable request.

## Abbreviations

GPs: General practitioners
