# Supplementary material for: Survey on the management of childhood epilepsy among general practitioners in the area of Marrakech
Source: BMC Pediatr. 2023 Apr 4;23:159. doi: 10.1186/s12887-023-03947-w (PMC10071602; doi:10.1186/s12887-023-03947-w)
Supplement: Supplementary file 1 — Additional file 1: Supplementary Material 1. [file 12887_2023_3947_MOESM1_ESM.docx]

**QUESTIONNAIRE**

Please check the answer(s) that you think correct(s)

1. Sector activity : Public Private Urban Rural
2. Duration of medical practice

- Less than 10 years
- More than 10 years

1. Sector of exercice

Private Health public center Hospital

1. After your medical course, have you received further training on the management of child’s epilepsy ?

Yes No

* If yes, please give more details about this experience :

…………………………………………………………………………………………………………………………………………………………………………………………………….

1. According to your knowledge, childhood epilepsy is a disease :

Rare Frequent Very frequent Benign Serious

1. The diagnosis of epilepsy is done on ?

- Single epileptic seizure Yes  No
- Repeated induced epileptic seizures Yes  No
- Repeated unprovoked epileptic seizures Yes  No

(* **induced** : provoked by Trigger factors such : stress, fever, infections,…)

1. How do you manage children with epilepsy in your daily practice ?

- Individually, without help of specialists Yes  No
- Asking for of specialists view Yes  No
- Systematically refer to specialists Yes  No

* If you refer to specialists, please give the reasons for this option :

……………………………………………………………………………………………………………………………………………………………………

1. How many cases per month of child epilepsy do you encounter in your workplace?

0 – 5 € 5 – 10 € > 10

1. Which age group do you think is most affected ?

€ < 2 years € 2 – 6 years € 6 – 12 years ≥ 12 years

1. For you, the diagnosis of epilepsy is based on :

Clinic only

Electroencephalogram (EEG) only

€ Electro-clinical

€ Clinical + EEG + Imaging

1. Do you request any other additional paraclinical exams ?

€ Yes € No

- If Yes, which ones ? : …………………………………………………………………………………..

1. According to your experience, what is the most common etiology ?

- Genetic factors
- Infectious diseases (Meningitis, Meningoencephalitis, ....)
- Brain tumors
- Cerebrovascular accident
- Cranial trauma
- Lesional (cerebral palsy, …)
- Others (Toxic, metabolic, drugs,…)
- Idiopathic

1. Do you rule out differential diagnoses before making a diagnosis of childhood epilepsy ?

€ Yes € No

* If Yes, which ones ? :

………………………………………………………………………………………

1. According to your experience, what are the seizures trigger factors ?

- Lack of sleep
- Hyperthermia
- Stress
- Hypoglycémie
- stimulating drinks (tea, coffee,…)
- Heat stroke
- Others : …………………………………………………………

1. Which type of epilepsy do you find most difficult to manage ?

- Partial
- Generalized

1. According to your experince, what are the most difficult syndromes to manage

- Tonico-clonic
- Myoclonic
- Atonic
- Absence epilepsy
- Infantile Spasms
- Others :…………………………………………………………………..

1. Have you ever diagnosed focal epilepsy in a child ?

€ Never € Rarely € Often € Very often

1. Duration of the persistent or repeated seizures necessary to make the diagnosis of status epilepticus in children is :

€ ≥ 5 min € ≥ 10 min € ≥ 15 min € ≥ 30 min

1. How do you manage status epilepticus in children?

- Individually without heilp of specialists
- Refer systematically to specialists

1. Do you now the last classifications of the ILAE «International League Against Epilepsy» ?

- Yes € No

1. Which therapeutic management do you adopt in first intention ?

€ Monotherapy € Dual therapy € Polytherapy

1. According to your experience, what is the duration of the antiepileptic treatment ?

6 months 12 months 2 years  5 years € for life

1. Which molecules do you prescribe as antiepileptic drugs?

- Sodium Valproate
- Diazepam
- Midazolam
- Phenobarbital
- Carbamazepine
- Lamotrigine
- Levetiracetam
- Others :……………………………………………………………………………

1. What principal factors influence your molecule’s choice ?

- Type of epilepsy  Yes  No
- Cost  Yes  No
- Efficiency  Yes  No
- Tolerance  Yes  No
- Others : …………………………………………………………………………

1. Do you look for side effects of molecules you prescribe ?

 Never  Rarely  Often  Always

1. Do you explain to the parents and their children the side effects of drugs you prescribe ?

€ Never € Rarely € Often Always

1. Do you follow up children after the first consultation?

€ Yes € No

* If yes : which rythm of control do you adopt?

- Once every 3 month
- Once every 6 month
- Once a year
- Others :………………………………….

1. According to your experience, it is necessary to provide psychological care for children with epilepsy ?

 Never  Often  Case by case  Always

1. According to your experience, it is necessary to provide psychological care for parents of children with epilepsy?

€ Never € Often € Case by case € Always

1. Do you give education for parents and their children with epilepsy ?

 Never  Often  Case by case  Always

1. What are your suggestions to improve management of children with epilepsy ?

……………………………………………………………………………………………………………………………………………………………………………………………………………………………………………………………………………………………………………………………………………………………………………………………………………………………………………………………………

Thank you for your attention and close collaboration
